# Clinical Evidence Linkage From the American Society of Clinical Oncology 2024 Conference Poster Images Using Generative AI: Exploratory Observational Study

**DOI:** 10.2196/78148

**Published:** 2026-02-05

**Authors:** Carlos Areia, Michael Taylor

**Affiliations:** 1 6th Briset street Digital Science (United Kingdom) London United Kingdom; 2 Coventry University Coventry United Kingdom; 3 University of Wolverhampton Wolverhamptom United Kingdom

**Keywords:** medical informatics, clinical decision support, generative artificial intelligence, oncology, conference posters, Altmetric, clinical trials, artificial intelligence, AI

## Abstract

**Background:**

Early-stage clinical findings often appear only as conference posters circulated on social media. Because posters rarely carry structured metadata, their citations are invisible to bibliometric and alternative metric tools, limiting real-time research discovery.

**Objective:**

This study aimed to determine whether a large language model can accurately extract citation data from clinical conference poster images shared on X (formerly known as Twitter) and link those data to the Dimensions and Altmetric databases.

**Methods:**

Poster images associated with the 2024 American Society of Clinical Oncology conference were searched using the terms “#ASCO24,” “#ASCO2024,” and the conference name. Images ≥100 kB that contained the word “poster” in the post text were retained. A prompt-engineered Gemini 2.0 Flash model classified images, summarized posters, and extracted structured citation elements (eg, authors, titles, and digital object identifiers [DOIs]) in JSON format. A hierarchical linkage algorithm matched extracted elements against Dimensions records, prioritizing persistent identifiers and then title-journal-author composites. Manual validation was performed on a random 20% sample.

**Results:**

We searched within 115,714 posts and 16,574 images, of which 651 (3.9%) met the inclusion criteria, and we obtained 1117 potential citations. The algorithm linked 63.4% (708/1117) of the citations to 616 unique research outputs (n=580, 94.2% journal articles; n=36, 5.8% clinical trial registrations). Manual review of 135 randomly sampled citations confirmed correct linkage in 124 (91.9%) cases. DOI-based matching was mostly flawless; most errors occurred where only partial bibliographic details were available. The linked dataset enabled rapid profiling of topical foci (eg, lung and breast cancer) and identification of the most frequently referenced institutions and clinical trials in shared posters.

**Conclusions:**

This study presents a novel artificial intelligence–driven methodology for enhancing research discovery and attention analysis from nontraditional clinical scholarly outputs. The American Society of Clinical Oncology was used as an example, but this methodology could be used for any conference and clinical poster.

## Introduction

### Background

Generative artificial intelligence (AI) has rapidly transformed image and visual processing, progressing from early generative adversarial networks to advanced multimodal models such as DALL-E and diffusion-based techniques. Recent studies highlight advancements in text-to-image generation and semantic image synthesis [1], showcasing improved realism and contextual understanding. The application of AI in visual communication design [2] and generative visual intelligence [3] further demonstrates AI’s expanding role in creative fields. As generative models continue to evolve, their impact extends beyond art and entertainment into scientific visualization and human-computer interaction.

Altmetric and Dimensions are two powerful research analytics platforms that provide insights into scholarly impact and research discovery. Altmetric specifically tracks the online attention a research output receives. It aggregates mentions from a wide variety of nontraditional sources, including social media (such as X, formerly known as Twitter, the platform used in this study), public policy documents, mainstream news outlets, podcasts, blogs, and others. This provides a real-time gauge of how research is being discussed and shared among both academic and public audiences, reflecting its societal or “alternative” impact [4]. Dimensions, on the other hand, is a comprehensive research database that integrates publications, grants, patents, clinical trials, and policy documents, enabling in-depth bibliometric analysis and research discovery [5]. Unlike traditional citation-based metrics, these platforms offer a broader perspective on research influence, making them an essential complement for researchers, institutions, and policymakers.

Extracting citations from research conference posters presents unique challenges due to their unstructured format, multimodal content, and limited metadata. Recent advances in scholarly document processing and bibliographic reference parsing have aimed to improve citation extraction from various scientific sources. Studies on neural network models for scholarly document processing [6] and automated bibliographic reference parsers provide insights into leveraging AI for structured citation extraction. Additionally, research on semantic entity extraction from academic databases [7] highlights challenges related to data acquisition and accuracy. These findings suggest that, while existing methods improve citation extraction in formal publications, further work is needed to adapt them for conference posters.

### Objectives

The primary goal of this study was to test whether it is possible to extract citations from conference poster pictures using a large language model (LLM). The secondary objective was to explore what extracted data can be used to link the poster citations to Dimensions and Altmetric data.

## Methods

### AI Prompt and Testing

To examine whether generative AI can extract information from conference posters, we used the Google Cloud Vertex AI environment and tested several LLMs. Through trial and error and comparison of the quality of the output, we used the Gemini 2.0 Flash (Google; experimental) version for this study. The prompt used is presented in Textbox 1.

**Figure 1:**
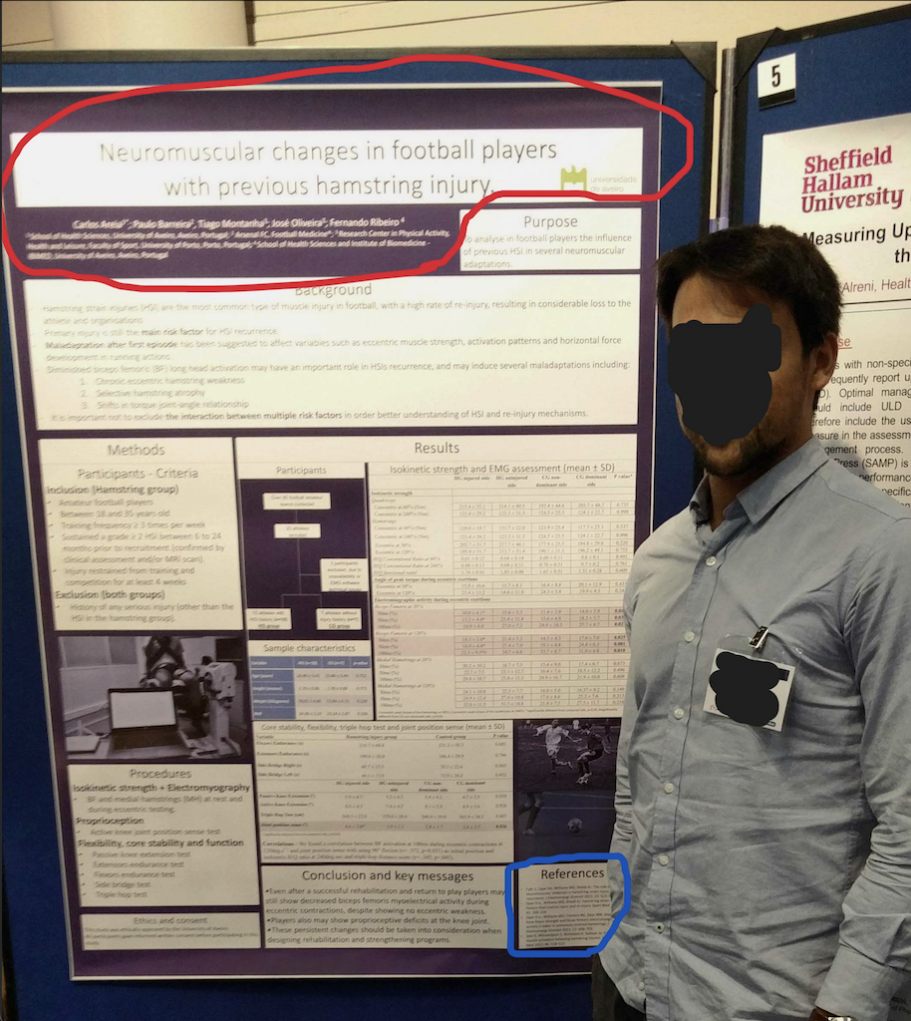
Example poster picture (with permission). The red outline represents extracted information from the main poster; the blue outline represents extracted information from references.

Figure 1 shows an example of a poster image with the locations from which information was extracted. The JSON format information returned by the prompt is provided in Textbox 2.

**Example prompt input.**

“You are a researcher and poster identification and citation reference extraction expert. I will provide you with the image or bucket location of a poster image and you are going to evaluate if the image:

1. is_research_poster (True/False)

2. image_type (one or more of these categories, separated by comma: people, poster, presentation_picture, presentation_slides, selfie, other)

3. image_type_other (if the above answer is other, add another category here)

4. is_readable (True/False)

5. poster_summary (100 words maximum)

If it is a poster then you are going extract the following information for each reference/citation mentioned in the research poster:

1. citation_n (0 is for the poster publication information itself, all the rest are citations)

2. authors

3. first_author_last_name

4. last_author_last_name

5. year

6. doi

7. identifier_other

8. journal_title_original

9. journal_title_full (full name of the journal)

10. volume

11. pages

12. title (just include the citation publication title text and nothing else)

13. other (include any other persistent identifier, link or information you see relevant to find the citation)

14. full_content (add everything included in the citation, for the main poster publication also add the affiliations, funders, sponsors and any other available information)

Go through these steps:

1- Confirm there is a research poster in the picture

2- If the image quality is low please focus on extracting the DOI at least 3 times. You can try to improve the quality of the pixels yourself

3- Identify the reference/citations section

4- Extract the information for each citation. Numbering each citation (citation #1, #2, etc...)

5- Only complete the fields if you are certain they are correct, otherwise respond null to that field

6- JSON format with all the fields detailed above”

**Extracted code.**

{

“is_research_poster”: true,

“image_type”: [

“poster”

],

“image_type_other”: null,

“is_readable”: true,

“poster_summary”: “This research poster investigates neuromuscular changes in football players with previous hamstring injuries (HSI). The study aims to analyze the influence of previous HSI on neuromuscular adaptations. The methods include isokinetic strength and EMG assessments, proprioception tests, and flexibility/core stability tests. The results show that even after rehabilitation, players may exhibit decreased biceps femoris myoelectrical activity during eccentric contractions and proprioceptive deficits at the knee joint. The conclusion emphasizes the need to consider these persistent changes in rehabilitation and strengthening programs.”,

“citations”: [

{

“citation_n”: 0,

“authors”: “Carlos Areia, Paulo Barreira, Tiago Montanha, José Oliveira, Fernando Ribeiro”,

“first_author_last_name”: “Areia”,

“last_author_last_name”: “Ribeiro”,

“year”: null,

“doi”: null,

“identifier_other”: null,

“journal_title_original”: null,

“journal_title_full”: null,

“volume”: null,

“pages”: null,

“title”: “Neuromuscular changes in football players with previous hamstring injury.”,

“other”: “1 School of Health Sciences, University of Aveiro, Aveiro, Portugal; 2 Arsenal FC, Football Medicine; 3 Research Center in Physical Activity, Health and Leisure, Faculty of Sport, University of Porto, Porto, Portugal; 4 School of Health Sciences and Institute of Biomedicine - İBİMED, University of Aveiro, Aveiro, Portugal”,

“full_content”: “Carlos Areia¹*; Paulo Barreira², Tiago Montanha³; José Oliveira³; Fernando Ribeiro 4\n1 School of Health Sciences, University of Aveiro, Aveiro, Portugal; 2 Arsenal FC, Football Medicine; 3 Research Center in Physical Activity, Health and Leisure, Faculty of Sport, University of Porto, Porto, Portugal; 4 School of Health Sciences and Institute of Biomedicine - İBİMED, University of Aveiro, Aveiro, Portugal”

},

{

“citation_n”: 1,

“authors”: “Fyfe JJ, Opar DA, Williams MD, Shield AJ”,

“first_author_last_name”: “Fyfe”,

“last_author_last_name”: “Shield”,

“year”: null,

“doi”: null,

“identifier_other”: null,

“journal_title_original”: null,

“journal_title_full”: null,

“volume”: null,

“pages”: null,

“title”: “The role of neuromuscular inhibition in hamstring strain injury”,

“other”: null,

“full_content”: “Fyfe JJ, Opar DA, Williams MD, Shield AJ. The role of neuromuscular inhibition in hamstring strain injury. Br J Sports Med 2013; 47: 86-92.”

},

{

“citation_n”: 2,

“authors”: “Opar DA, Williams MD, Shield AJ”,

“first_author_last_name”: “Opar”,

“last_author_last_name”: “Shield”,

“year”: “2012”,

“doi”: null,

“identifier_other”: null,

“journal_title_original”: null,

“journal_title_full”: null,

“volume”: “42”,

“pages”: “209-224”,

“title”: “Hamstring strain injuries: factors that lead to injury and re-injury”,

“other”: null,

“full_content”: “Opar DA, Williams MD, Shield AJ. Hamstring strain injuries: factors that lead to injury and re-injury. Sports Med 2012; 42: 209-224”

},

{

“citation_n”: 3,

“authors”: “Brockett CL, Morgan DL, Proske U”,

“first_author_last_name”: “Brockett”,

“last_author_last_name”: “Proske”,

“year”: “2004”,

“doi”: null,

“identifier_other”: null,

“journal_title_original”: null,

“journal_title_full”: null,

“volume”: null,

“pages”: null,

“title”: “Human hamstring muscles adapt to eccentric exercise by changing contraction-induced injury”,

“other”: null,

“full_content”: “Brockett CL, Morgan DL, Proske U. Human hamstring muscles adapt to eccentric exercise by changing contraction-induced injury. Med Sci Sports Exerc 2004; 36: 379-383.”

},

{

“citation_n”: 4,

“authors”: “Schache AG, Blanch P, Rath D, et al”,

“first_author_last_name”: “Schache”,

“last_author_last_name”: “al”,

“year”: “2011”,

“doi”: null,

“identifier_other”: null,

“journal_title_original”: null,

“journal_title_full”: null,

“volume”: “46”,

“pages”: “118-121”,

“title”: “Hamstring muscle strength and flexibility in elite Australian Rules football players with previous hamstring strain injury”,

“other”: null,

“full_content”: “Schache AG, Blanch P, Rath D, et al. Hamstring muscle strength and flexibility in elite Australian Rules football players with previous hamstring strain injury. J Sci Med Sport 2011; 46: 118-121”

}

]

### Poster Image Extraction and Inclusion Criteria

For the purposes of this study, we selected poster images posted on X associated with the American Society of Clinical Oncology (ASCO) 2024 conference. We used the Tweepy Python library [8] to do this using the following search terms: “American Society of Clinical Oncology Annual Meeting 2024” OR #ASCO24 OR #ASCO2024

Due to the high metadata availability of ASCO conferences, we also decided to include a smaller, nonclinical conference to double-check accuracy and citation linkage. For this subanalysis, we included all International Conference on Science, Technology, and Innovation Indicators (STI) conferences from 2018 to 2024 (Multimedia Appendix 1).

For the image to be considered for inclusion, it had to be at least 100 kB and mention the word “poster” in the body of the post to optimize the identification of posts that contained a poster image to input into the LLM.

### Conference Image Analysis at Scale

**Figure 2:**
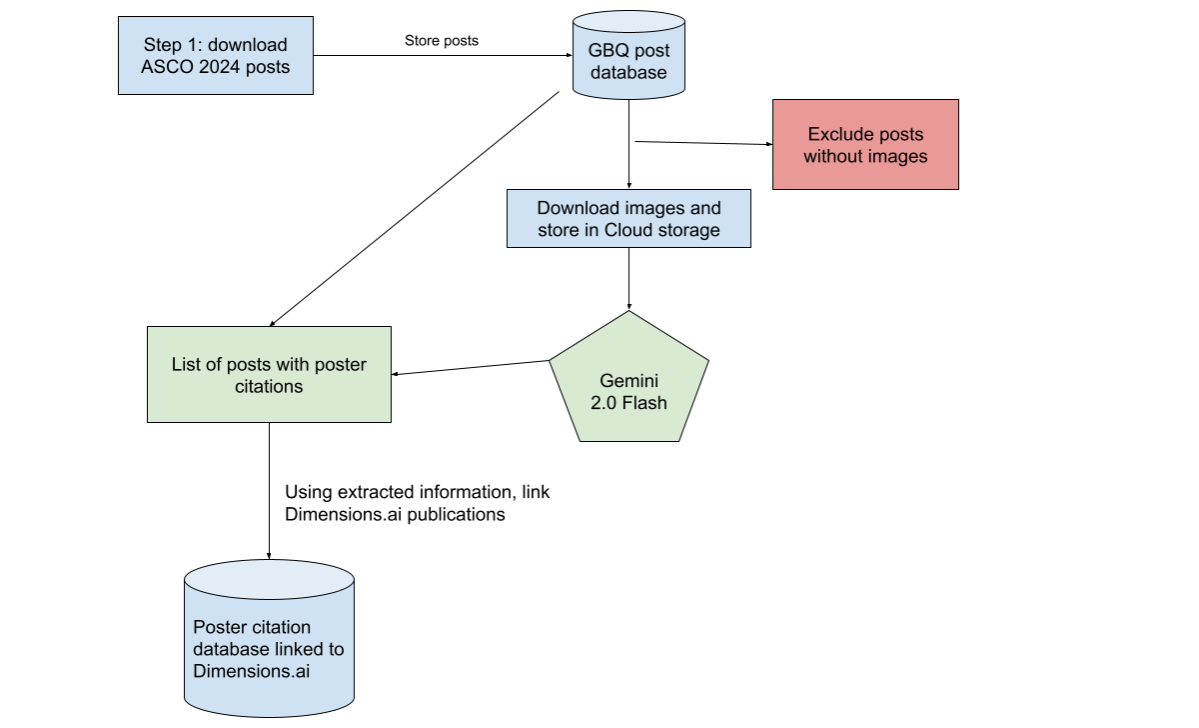
Image analysis and linkage pipeline. ASCO: American Society of Clinical Oncology; GBQ: Google BigQuery.

To be able to do this at scale, we used the following toolkits: Python (Python Software Foundation); Jupyter Notebooks; and Google Cloud Vertex AI, Cloud Storage, and BigQuery. The pipeline is described in Figure 2.

### Linkage Algorithm

From the AI response containing the JSON values, we extracted and linked data to our Dimensions database using an algorithm that considered the following information:

- Digital object identifier (DOI) or any other persistent identifier: this was the most straightforward method of linkage; when a persistent identifier was available, we used it to directly link it with the Dimensions database
- First author last name
- Last author last name
- Year
- Journal
- Volume
- Pages
- Title matching

Title matching involved 2 calculations. The first was match percentage to identify the textual overlap between citation and publication titles while accounting for differences in word counts. First, titles were tokenized to calculate word counts and matching words. A match percentage was derived by dividing matching words by the citation title word count. To address noise from large word count discrepancies, an adjustment penalized cases with significant differences, weighting matches in which word counts aligned more closely.

The second calculation was title score=match percentage×number of words. This was also calculated to differentiate strong matches in low-count titles that could be present in many different publications due to commonality.

The primary and simplest method for matching were the persistent identifiers. The next option was the title matching algorithm, where only matches with an adjusted match percentage above 70% were considered for use, with those with a percentage of >93% used on its own and those with a percentage between 70% and 90% used in conjunction with the above indicators. Table 1 describes the algorithm used by priority order.

**Table 1:** Matching method priority table.^a^

Matching method	Match percentage	Other matches used
DOI	—^b^	—
PMID	—	—
Clinical trial registry	—	—
Title_Only	93% 82% (+title score >7) 71% (+title score >15)	—
Title_Year_Journal	70%	Journal title Citation/publication year
Title_First_Author	70%	First author last name
Title_Last_Author	70%	Last author last name
Title_Volume_Pages	62%	Journal volume Journal pages
Title_Authors	100% (exact match)	First author last name Last author last namec
Journal_Volume_Pages	—	Journal title Journal volume Journal pages
Journal_Pages_Year	—	Journal title Journal pages publication year
Journal_Year_Author	—	Journal title Publication year First or last author last name

^a^The “authors only” method was used due to our single-conference, small-sample example. This should not be used at scale as it might match a high number of wrong publications (same authors, different studies).

^b^Not used.

^c^There need to be two of the following matches: first to first, last to last, first to last, or last to first.

In the case of multiple Dimensions publication matches, we used the highest match percentage or title score. After all these algorithms had run, we retrieved all the Dimensions IDs linked to DOI, PubMed identifier (PMID), clinical trial registration, title, and journal. For the final ID matching decision, we applied the following rules (see the example in Table 2). If multiple IDs were retrieved, we selected the most frequent ID as the final ID. If only 1 ID was retrieved, that was the final ID. If multiple IDs were retrieved but not repeated, we used the following priority list: (1) retrieved DOI, (2) retrieved PMID, (3) retrieved clinical trial ID, (4) retrieved title ID, and (5) retrieved journal ID.

**Table 2:** Algorithm prioritization example using fictional IDs.

Digital object identifier	PubMed identifier	Trial ID	Title ID	Journal ID	Final ID
pub.1234567	pub.1111111	—^a^	pub.1111111	—	pub.1111111
pub.1234567	—	—	—	—	pub.1234567
—	pub.1111111	—	—	—	pub.1111111
—	—	NCT882929	—	—	NCT882929
—	—	—	—	pub.123123	pub.123123
pub.1234567	pub.1111111	—	—	—	pub.1234567
—	—	—	pub.1111111	pub.123123	pub.1111111

^a^Not used.

### Random Sampling Check

To test the accuracy of the citation extraction and linkage, we performed a random check of examples, first, for whether the citation was correct regarding the image information. If this information was false, we checked whether it was the fault of the AI extraction (eg, extracting the wrong DOI and hallucinating an identifier) and whether it was the fault of the matching algorithm (eg, when the AI extracted the information correctly but we could not match it to our Dimensions and Altmetric data).

To ensure that we had at least 100 manually confirmed citations, we randomly selected 150 examples from our results dataset. For our subanalysis, due to the small sample size, all citations were manually confirmed (Multimedia Appendix 1).

### Ethical Considerations

Due to the observational nature of publicly available data, this study was exempt from ethics approval and informed consent. The example image of the poster in Figure 1 is from the main author of this manuscript and is shared with his permission.

## Results

### Overview

This study included, in total, 115,714 X posts from the ASCO 2024 conference, including 23,548 (20.4%) original posts, 4044 (3.5%) quoted posts, and 88,122 (76.2%) reposts. Of these 115,714 posts, 18,218 (15.7%) included at least one attachment, with 16,574 (14.3%) being labeled as a photo or image. After applying our eligibility criteria (image of >100 kB and text of the post including the word “poster”), we narrowed this down to 793 images to run our AI model. Of these 793 images, a further 94 (11.9%) were excluded from the analysis as the quality was too low to be readable by the AI, and 130 (16.4%) were considered posters by the AI, all confirmed manually (Figure 2). The final dataset included 651 readable poster images, with 1117 potential citations identified by our AI model.

**Figure 3:**
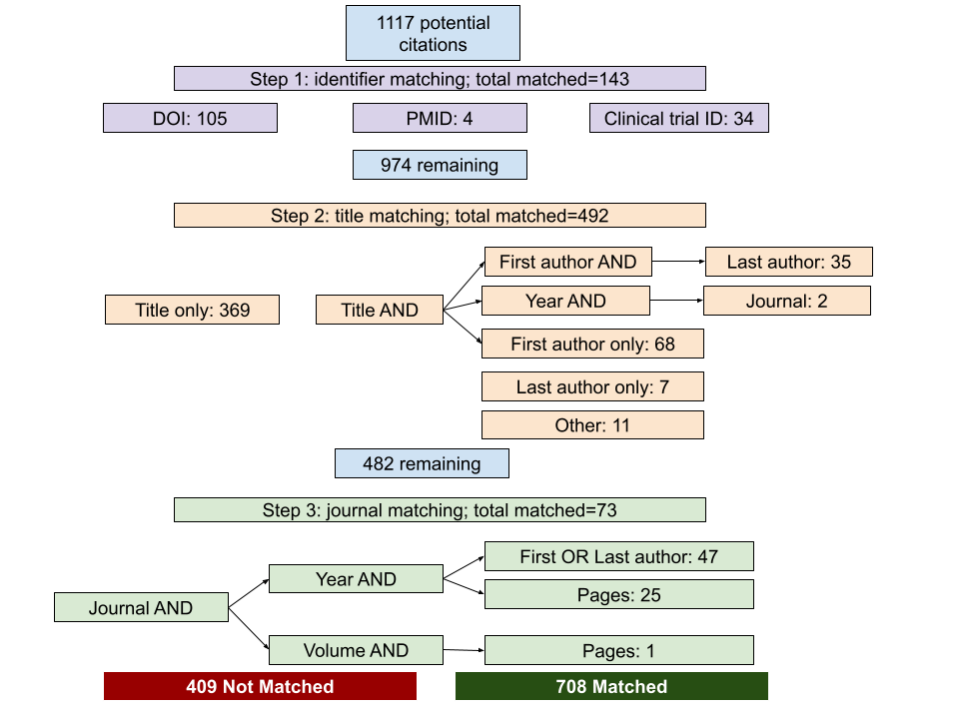
Poster citation to publication matching algorithm process and results. DOI: digital object identifier; PMID: PubMed identifier.

In total, we managed to link 708 poster citations (n=332, 46.9% coming from the main poster information and n=376, 53.1% from the reference section or content of the poster, with an average of 1.66, SD 2.58 and median of 1, IQR 0-3 references per poster) to their respective publication information (616 unique publications) using different matching methods as described in Figure 3.

We were unable to link over one-third (409/1117, 36.6%) of the potential citations to their respective Dimensions publication information due to incomplete information or to the information retrieving multiple studies, as in the following examples:

- Cox AD et al
  Nat Rev Drug Discov
  (insufficient information)
- Accurate detection of ER loss (
  J Clin Oncol
  2022) (unable to match any publication)
- Schuler et al, 2024 (matching multiple publications)
- https://www.cancer.org/cancer/types/prostate-cancer/about/key-statistics (not a citation)
- NURE-COMBO trial: NCT04086115 (identifier outdated or not correct)
- (Abstract #10521, Hedin T et al.) (not indexed abstract and/or publication)

Our subanalysis results can be found in Multimedia Appendix 1.

### Validation Accuracy Testing

Of the 708 matches, 150 (21.2%) were randomly selected for manual curation and confirmation of accuracy. Of these, after excluding the posters for which we were unable to confirm a match (not of sufficient quality to be readable by the human eye), we manually reviewed 135 poster citations, of which 124 (91.9%) were correct matches and 11 (8.1%) were incorrect matches. A breakdown by matching method can be found in Table 3.

Our subanalysis of the STI conferences yielded similar accuracy results (Multimedia Appendix 1).

**Table 3:** Number of accurate and inaccurate matches by method (N=135).

Matching method	Matched?	Matches, n (%)
Title and other	Yes	2 (1.5)
Title and other	No	1 (0.7)
Title only	Yes	76 (56.3)
Title only	No	3 (2.2)
Title and last author	Yes	2 (1.5)
Title and first author	No	2 (1.5)
Title and first author	Yes	6 (4.4)
Title and authors	Yes	8 (5.9)
PubMed identifier	Yes	1 (0.7)
Journal, year, and author	No	1 (0.7)
Journal, year, and author	Yes	9 (6.7)
Journal, pages, and year	Yes	4 (3.0)
Digital object identifier	No	4 (3.0)
Digital object identifier	Yes	9 (6.7)
Clinical trial registry identifier	Yes	7 (5.2)

### Included Publication Information

A total of 616 unique research outputs (n=580, 94.2% articles and n=36, 5.8% clinical trial registrations) were matched, with a few being mentioned in more than one conference poster (Table 4).

**Table 4:** Top 10 matched publications by the number of poster citations.

Year	Journal	Publication title	Poster citations, n	Study
2024	*Journal of Clinical Oncology*	“The genomic, transcriptomic, and immunological profile of patients with recurrent/refractory NSCLC”	3	[9]
2024	*Journal of Clinical Oncology*	“Exploring T cell subsets as predictors of response to BCMA targeting bispecific antibody therapy in multiple myeloma”	3	[10]
2024	*Journal of Clinical Oncology*	“Association between circulating tumor DNA (ctDNA) and recurrence-free survival (RFS) in patients (pts) with resected stage III melanoma: an exploratory analysis of SWOG S1404”	3	[11]
2024	*Journal of Clinical Oncology*	“Challenges and solutions to recruiting diverse populations to oncology clinical trials: a mixed-methods study of clinical research coordinators”	3	[12]
2024	*Journal of Clinical Oncology*	“Unveiling inequities in representation: racial disparities in supportive care breast cancer clinical trial enrollment”	3	[13]
2024	*Journal of Clinical Oncology*	“Final results of CORE-001: a phase-2, single arm study of cretostimogene grenadenorepvec in combination with pembrolizumab in patients with BCG-unresponsive, non-muscle invasive bladder cancer with carcinoma in situ”	3	[14]
2024	*Journal of Clinical Oncology*	“AI-based approach to enable proactive identification of early lung cancer: a retrospective population health study and economic model”	3	[15]
2024	*Journal of Clinical Oncology*	“First-line systemic therapy following adjuvant immunotherapy in renal cell carcinoma (RCC): an international multi-center study”	2	[16]
2024	*Journal of Clinical Oncology*	“Self-expressed needs and gaps in our care of metastatic breast cancer (MBC): an all-Ireland patient-led online survey (CTRIAL-IE 23-05)”	2	[17]
2020	*Annals of Oncology*	“PALLAS: A randomized phase III trial of adjuvant palbociclib with endocrine therapy versus endocrine therapy alone for HR+/HER2- early breast cancer”	2	[18]

The 616 research outputs linked as per Dimensions document classification [5] included 361 (58.6%) conference abstracts, 143 (23.2%) research articles, 39 (6.3%) review articles, 36 (5.8%) clinical trial registrations, 13 (2.1%) letters to the editor, 10 (1.6%) unknown, 4 (0.6%) other types of journal content, 4 (0.6%) correction or erratum notes, 3 (0.5%) editorials, 1 (0.2%) conference paper, 1 (0.2%) other type of conference content, and 1 (0.2%) reference work. Interestingly, some of the main posters were then published (either as an abstract or full publication) and achieved a significant level of attention (according to the Altmetric score), as shown in Table 5.

**Figure 4:**
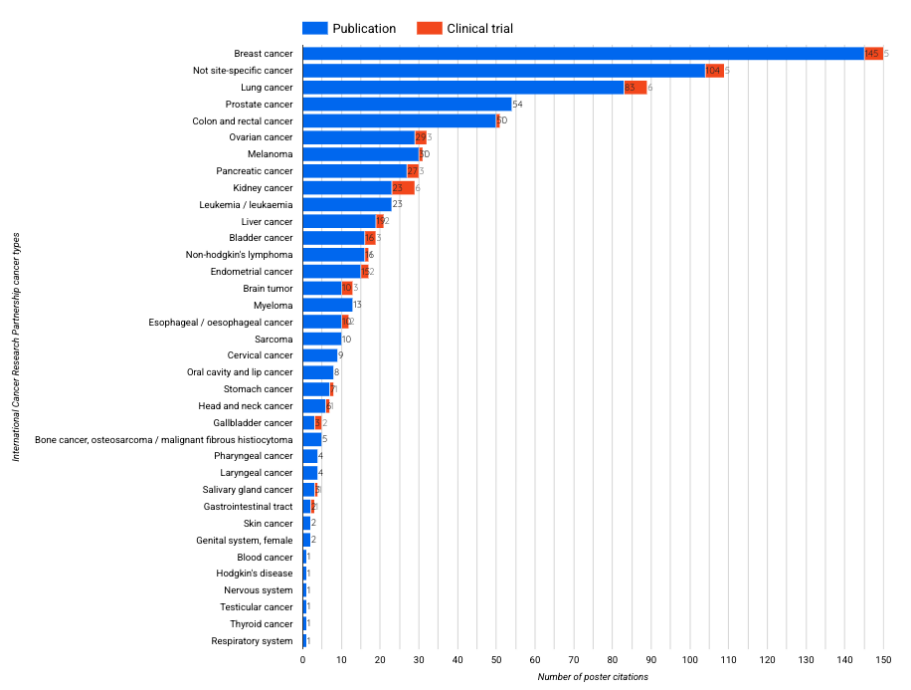
Top cancer type research mentioned in the American Society of Clinical Oncology 2024 conference posters classified by publication and clinical trial citation according to the International Cancer Research Partnership cancer type taxonomy.

By linking the poster citations to Dimensions publication and clinical trial information, it is possible to conduct several analyses and extract interesting signals, for example, using research categories. In Figure 4, we used the International Cancer Research Partnership cancer type taxonomy to be able to quickly identify the main cancer types mentioned in the poster citations.

**Table 5:** Top 10 published posters by Altmetric score.

Year	Journal	Publication title	Altmetric score	Study
2024	*Journal of Clinical Oncology*	“Outcomes of myeloma cast nephropathy in the era of anti-CD38 monoclonal antibody-based frontline therapy: A retrospective cohort study”	296	[19]
2024	*JCO Clinical Cancer Informatics*	“Characterizing the increase in artificial intelligence content detection in oncology scientific abstracts from 2021 to 2023”	65	[20]
2024	*Journal of Clinical Oncology*	“Results from the randomized phase III DREAMM-7 study of belantamab mafodotin (belamaf) + bortezomib, and dexamethasone (BVd) vs daratumumab, bortezomib, and dexamethasone (DVd) in relapsed/refractory multiple myeloma (RRMM)”	43	[21]
2024	*Journal of Clinical Oncology*	“Zanidatamab in previously-treated HER2-positive (HER2+) biliary tract cancer (BTC): Overall survival (OS) and longer follow-up from the phase 2b HERIZON-BTC-01 study”	39	[22]
2024	*European Urology*	“First-line systemic therapy following adjuvant immunotherapy in renal cell carcinoma: an international multicenter study”	30	[23]
2024	*Journal of Clinical Oncology*	“Inpatient burden and clinical outcomes of cytokine release syndrome in patients with cancer: a National Inpatient Sample study”	20	[24]
2021 (trial start date)	*ClinicalTrials.gov*	“A Phase 1b trial of M3814 (peposertib) in combination with lutetium 177 dotatate for Well-differentiated somatostatin receptor-positive gastroenteropancreatic neuroendocrine tumors (GEP-NETs)”	19	[25]
2024	*Journal of Clinical Oncology*	“Randomized study to assess colonic microbiome changes in response to energy drink consumption (ROSANNA trial)”	18	[26]
2024	*Journal of Clinical Oncology*	“Atezolizumab versus placebo in combination with bevacizumab and non-platinum-based chemotherapy in recurrent ovarian cancer: final overall and progression-free survival results from the AGO-OVAR 2.29/ENGOT-ov34 study”	18	[27]
2024	*Journal of Clinical Oncology*	“Self-expressed needs and gaps in our care of metastatic breast cancer (MBC): an all-Ireland patient-led online survey (CTRIAL-IE 23-05)”	13	[17]

We then used the authors’ affiliations to calculate the most frequently referenced institutions in shared posters (this could include the poster itself or inside reference), with the following top three for research articles: (1) Dana-Farber Cancer Institute (35 poster citations), (2) the University of Texas MD Anderson Cancer Center (30 poster citations), and (3) Memorial Sloan Kettering Cancer Center (24 poster citations).

The following were the top three for clinical trials: (1) SWOG Cancer Research Network (4 poster citations), (2) University of North Carolina Lineberger Comprehensive Cancer Center (3 poster citations), and (3) National Cancer Institute (3 poster citations).

**Figure 5:**
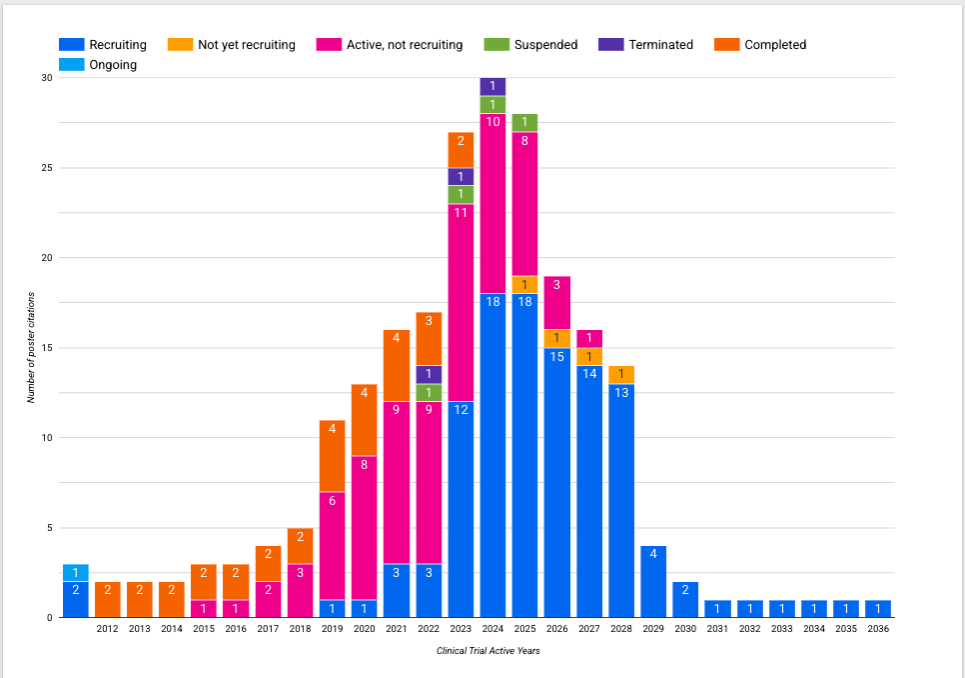
Overall status and active years of all clinical trials cited by the American Society of Clinical Oncology 2024 conference posters.

**Figure 6:**
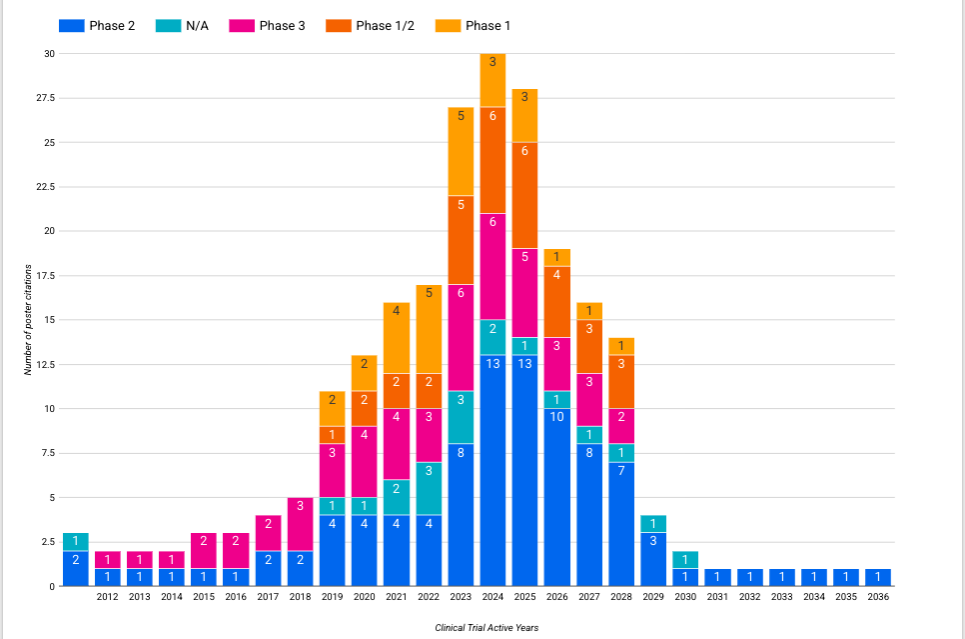
Trial phase and active years of all clinical trials cited by the American Society of Clinical Oncology 2024 conference posters. N/A: not applicable.

Focusing on clinical trials, by linking cited clinical trials to their respective Dimensions information, we were able to explore some interesting information and signals on the status of these trials; for example, in Figures 5 and 6, we outline the active years and overall status and phase for all 36 matched clinical trial registrations.

### Included X Profiles and Posts

The final dataset included 651 images with readable posters and 1117 potential citations. These images came from 240 X profiles and 347 unique X posts, ranging from a minimum of 1 to a maximum of 4 images per post.

**Figure 7:**
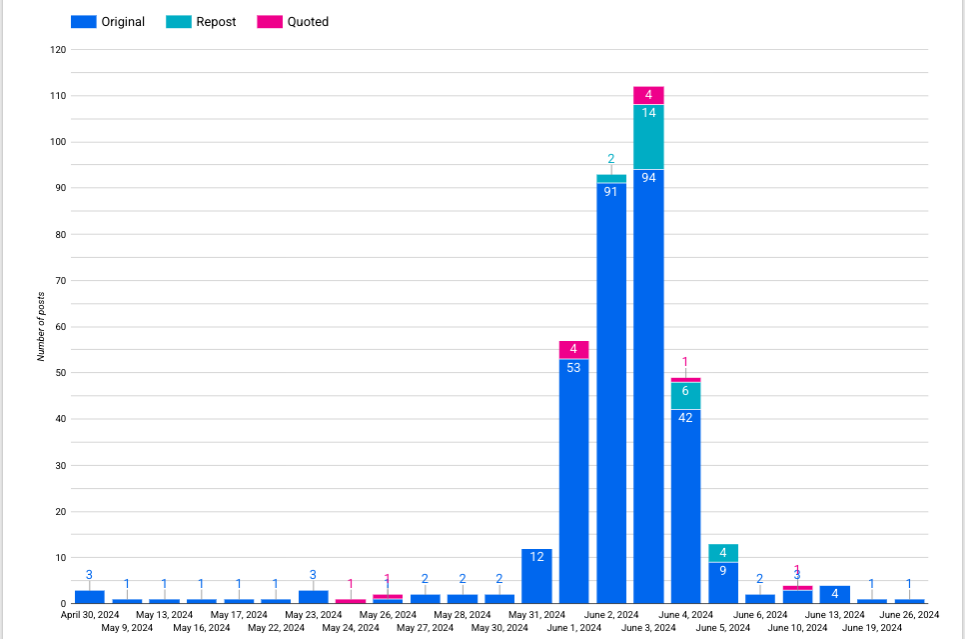
Included post timeline classified by type of post.

Most of the included posts (330/347, 95.1%) were original posts, with most (318/347, 91.6%) originating during the ASCO 2024 conference period (May 31, 2024, to June 4, 2024), as shown in Figure 7.

The top three profiles sharing posters were the following: (1) DFCI_BreastOnc (14 posts with 16 poster images), (2) CSCancerCenter (7 posts with 7 poster images), and (3) MDAndersonNews (6 posts with 6 poster images).

## Discussion

### Principal Results

This study aimed to explore the use of an LLM to extract both the main citations as well as references (where available) from poster images. It also explored how were different strategies good at trying to link the free-text extracted poster citation data to the Dimensions and Altmetric databases. As the ASCO conferences are well indexed, mostly in the *Journal of Clinical Oncology*, we decided to also include a subanalysis of a smaller, nonclinical conference that matched similar results. The LLM and algorithm can be tested on any poster at any conference.

This exploratory study demonstrated that it is indeed possible to extract structured citation data from poster images using LLMs and relatively easy to link to the Dimensions and Altmetric databases in cases in which the DOI or other persistent identifiers are present; however, it became significantly more challenging for references without these identifiers, and we tried to find creative solutions using the available extracted data points. Our random sampling accuracy testing highlighted that the LLM was particularly good at extracting the correct information from both the main poster and references (or other poster content citations), with the former usually being easier to link to the publication through the title and authors. When an identifier was provided in the references section, it was also quite straightforward to link it to the respective publication; however, it was more challenging to do so when only limited information was provided, for example, just the first author’s last name with “et al” and the journal (with the year in parentheses) as it could provide erroneous linkage to multiple publications.

For most posters (124/135, 91.9%), the LLM extracted the correct information, and using our Dimensions database, we managed to successfully link most of the citations with complete data provided in the poster. In some cases, we even managed to link a poster to both its respective clinical trial registration [28] and publication [29]. Interestingly, during the manual random sample, in a lot of cases in which a picture was taken of several posters and people at the same time, the AI still managed to extract enough partial information to be able to link to the publication data. According to our random sampling analysis, most of the errors were not from the AI extraction but from our matching algorithm, and several future improvements were identified. First, journal+year+volume is not sufficient and should only be used as a last resort. Many of the errors encountered were in this category, and despite this being already one of the most recent matching methods within our algorithm, we should consider other, more recent methods before this one. Second, even when using identifiers, we should consider other methods of matching in conjunction with them. In some cases, the AI seemed to hallucinate a DOI that matched the wrong publication. In other cases, the DOI was wrong (we noticed this particularly in older studies).

Our matching algorithm was relatively basic and needs further improvement as it was built through trial and error until an optimal level of accuracy was achieved using the selected method. Albeit limited, this algorithm was sufficient to prove our hypothesis successfully. In future studies and applications of this technology, we will consider other matching methods such as using multiple types of author information, sponsors, funders, organizations, and the content of the poster itself (which we can try to match to an abstract, for example). However, to the best of our knowledge, this is the first study to successfully extract poster image information and link it to a research repository. Despite our hypothesis’s success, poster citations should be used thoughtfully to avoid citation duplication (eg, if the conference poster abstract is published online). Therefore, it is our view that these poster citations may not be suitable to be used as formal citations but should perhaps be considered as attention mentions instead (a debatable opinion). As the images were shared through an X post, we believe that the correct way of presenting this information would be as a research attention (similar to when someone posts a publication link or DOI), and this would be a novel way to track conference attention.

Alternative metrics are becoming increasingly important, and tools such as Altmetric, PlumX, and others are increasing their capabilities in capturing different types of research attention [30]. Conference posters can contain novel, important information that often goes unpublished or share early insights on studies (eg, interim clinical trial results) months or years before the final publication [31]. We used the ASCO 2024 conference example to highlight that, due to the agile nature of health care practice (see the COVID-19 pandemic as an example), having access to timely poster citation information may be important for clinical and strategic decisions.

By linking poster citations to their respective research publication or clinical trial information, it is possible to extract interesting insights and signals to explore at scale. In this study, we used a narrow, limiting example as a proof of concept; however, future research will test this at scale with multiple conferences through the years in several fields (including new ones such as humanities, social sciences, and engineering), testing practical, economical, and computational feasibility of poster or image citation extraction.

### Limitations

We noticed in some rare cases that, if a poster was about a systematic review that referenced other publications in the content, the LLM often assumed that to be a poster mention. While not the desired behavior, it might extend the capabilities of this model and the number of publications identified as cited in that poster (which is, in fact, correct and in accordance with our study objectives).

ASCO posters are routinely published by the *Journal of Clinical Oncology* and issued a DOI, and therefore, the metadata are easy to obtain. Many other important conferences do not publish material with DOIs and would be considerably harder to match. To test this, we used a smaller nonclinical conference as a subanalysis (Multimedia Appendix 1) that yielded similar results.

During the random sampling accuracy testing, we noted that, when the quality of the poster was poor but passable (according to the *is_readable* variable returned by the AI), it was hard for the human eye to confirm its accuracy, and therefore, the poster was not included in the random sampling analysis. Future work should include thresholds of quality for accuracy checking. We also did not perform a sample size calculation for the random sampling analysis, and the 150-sample check was an arbitrary number that we felt was sufficient to be representative of our data together with the STI conference subanalysis, in which we conducted the accuracy check in all citations due to the small sample.

Another limitation is that we did not fully explore linking methods between the data retrieved from the image and our datasets. There might be other creative ways to link more publications to our (and other) data. For example, in this study, we struggled to match publications in which only the first author, year, and journal were shared, and there might be other clever ways to match these in our dataset (by increasing context using general poster information and perhaps another AI model to help select the correct publication within a list). Another example is when the LLM hallucinated while extracting a persistent identifier (eg, DOI or PMID) and other information was provided. A fallback method could be added to the algorithm to identify a nonmatch from the identifier and prioritize other matching methods instead. Future work will involve improving our algorithm to maximize poster citation matching.

This study also opens the door to other image citation extraction algorithms, for example, of conference presentations (or available online presentation slides in which research mention and citation is likely) or presentation video analysis and citation extraction using AI.

### Conclusions

The objective of this study was to confirm the hypothesis that AI can be used to extract citations from poster images. Our study concluded that it is not only possible but also straightforward to do it in a scalable way, with most of the effort lying in accurately connecting these citations to the correct publication data through different methods. This study opens the door for future use of AI on image data extraction to collect scholarly mentions and citations from novel sources, as well as other relevant clinical data from conference posters.

## Appendix

Multimedia Appendix 1 International Conference on Science, Technology, and Innovation Indicators subanalysis.

## Abbreviations

AI: artificial intelligence
ASCO: American Society of Clinical Oncology
DOI: digital object identifier
LLM: large language model
PMID: PubMed identifier
STI: International Conference on Science, Technology, and Innovation Indicators

## Data Availability

Some datasets generated or analyzed during this study are available from the corresponding author on reasonable request.
