# Supplementary material for: Clinical Evidence Linkage From the American Society of Clinical Oncology 2024 Conference Poster Images Using Generative AI: Exploratory Observational Study
Source: JMIR AI. 2026 Feb 5;5:e78148. doi: 10.2196/78148 (PMC12921429; doi:10.2196/78148)
Supplement: Multimedia Appendix 1 [file ai_v5i1e78148_app1.docx]

# **Multimedia Appendix 1 - STI conferences subanalysis**

## **Search terms used**

- "International Conference on Science, Technology and Innovation Indicators 2024" OR #STI2024 OR #STI2024Berlin OR @sti2024Berlin OR from:sti2024Berlin
- "International Conference on Science, Technology and Innovation Indicators 2023" OR #STI2023 OR @sti2023 OR from:sti2023
- "International Conference on Science, Technology and Innovation Indicators 2022" OR #STI2022 OR #STI2022grx OR @sti2022grx OR from:sti2022grx
- "International Conference on Science, Technology and Innovation Indicators 2021" OR #STI2021 OR #STI2021Aarhus OR @STI2021Aarhus OR from:STI2021Aarhus
- "International Conference on Science, Technology and Innovation Indicators 2019" OR #STI2019 OR #ISSI2019
- "International Conference on Science, Technology and Innovation Indicators 2018" OR #STI2018 OR #STI18LDN

## **Results**

| **Matching method** | **Matched?** | **Number** | **Percentage** |
| --- | --- | --- | --- |
| Title_Only | Yes | 28 | 63.64% |
| Title_Only | No | 3 | 6.82% |
| Title_First_Author | Yes | 4 | 9.09% |
| Title_First_Author | No | 1 | 2.27% |
| Title_Authors | Yes | 5 | 11.36% |
| Title_Authors | No | 1 | 2.27% |
| Journal_Year_Author | Yes | 1 | 2.27% |
| DOI | Yes | 1 | 2.27% |
